# Joint line convergence angle is the most associated alignment factor with the severity of medial knee osteoarthritis

**DOI:** 10.1002/jeo2.70007

**Published:** 2024-08-22

**Authors:** Takahiro Tsushima, Eiji Sasaki, Yukiko Sakamoto, Yuka Kimura, Eiichi Tsuda, Yasuyuki Ishibashi

**Affiliations:** ^1^ Department of Orthopaedic Surgery Hirosaki University, Graduate School of Medicine Hirosaki Japan; ^2^ Department of Rehabilitation Medicine Hirosaki University, Graduate School of Medicine Hirosaki Japan

**Keywords:** cartilage degeneration, joint line convergence angle, Kellgren–Lawrence grade, medial knee osteoarthritis, medial meniscus extrusion

## Abstract

**Purpose:**

The purpose of this study was to evaluate the relationship between the joint line convergence angle (JLCA) and the severity of medial knee osteoarthritis (OA). We hypothesise that JLCA is the most associated factor with the severity of medial knee OA.

**Methods:**

This retrospective study included a total of 202 knees that underwent either high tibial osteotomy or medial meniscus repair/partial resection. Kellgren–Lawrence grade and hip–knee–ankle angle (HKAA), mechanical lateral distal femoral angle (mLDFA), medial proximal tibial angle (MPTA) and JLCA were assessed from preoperative radiographs. Medial meniscus extrusion (MME) was measured using preoperative magnetic resonance imaging. The International Cartilage Research Society (ICRS) grade on the medial femoral condyle and medial tibial plateau were also assessed. The relationships between JLCA and Kellgren–Lawrence grades and MME and ICRS grades were analysed using Spearman's correlation test and regression analysis.

**Results:**

The JLCA was correlated with the Kellgren–Lawrence grade (*R* = 0.765, *p* < 0.001), MME (*R* = 0.638, *p* < 0.001), ICRS grade on the MFC (*R* = 0.586, *p* < 0.001) and the MTP (*R* = 0.586, *p* < 0.001). Regression analysis showed that age (*p* = 0.002) and JLCA (*p* < 0.001) were associated with Kellgren–Lawrence grade. Furthermore, JLCA was related to ICRS grade on the MFC (*p* < 0.001) and MTP (*p* < 0.001).

**Conclusion:**

The JLCA, reflecting radiological severity, meniscus status, and cartilage lesion, was the most associated alignment parameter in the severity of medial knee OA. The JLCA may be beneficial for quantitative assessment of medial knee OA.

**Level of Evidence:**

Level III, retrospective cohort study.

**Abbreviations**
BMI: body mass index
CI: confidence interval
HKAA: hip–knee–ankle angle
ICRS: International Cartilage Research Society
JLCA: joint line convergence angle
MFC: medial femoral condyle
mLDFA: mechanical lateral distal femoral angle
MME: medial meniscus extrusion
MPTA: medial proximal tibial angle
OA: osteoarthritis
OWHTO: medial open wedge high tibial osteotomy
ROC: receiver operating characteristics
TKA: total knee arthroplasty

## INTRODUCTION

Osteoarthritis (OA) of the knee is a degenerative disease that gradually progresses, impacting activities of daily living and quality of life, primarily due to knee pain [34]. The surgical options for medial knee OA include open wedge high tibial osteotomy (OWHTO) [2, 5, 11, 17, 25], unicompartmental knee arthroplasty (UKA) [21, 27, 31] or total knee arthroplasty (TKA) [10, 12, 27] and the decision requires an adequate assessment. The joint line convergence angle (JLCA) reflects joint laxity [15, 24, 29], meniscus tear [14] and OA progression [3, 6, 19, 32] of the knee. However, the relevance of JLCA to radiological OA progression, meniscus condition and arthroscopic severity of cartilage degeneration in the medial knee OA has not been quantitatively or statistically assessed in previous reports. This study aimed to assess the relationship between JLCA and radiological severity, meniscus condition and arthroscopic cartilage degeneration in patients with medial knee OA. It was hypothesised that JLCA related to Kellgren–Lawrence grade, meniscus condition and cartilage degeneration, is the most associated factor with the severity of medial knee OA. If the severity of medial knee OA can be quantitatively assessed using JLCA on radiographs and predicted articular cartilage status, it would help orthopaedic surgeons decide whether to perform OWHTO or UKA for medial knee OA. It is known that the clinical results of OWHTO for patients with severe knee OA are inferior to those for patients with early knee OA [33]. Therefore, the indications for UKA might be considered for patients with severe knee OA.

## MATERIALS AND METHODS

### Patients

This retrospective study was conducted from January 2010 to January 2022. A total of 202 knees, including 141 knees that underwent medial OWHTO, 49 knees that underwent medial meniscus repair and 12 knees that underwent medial meniscus partial resection for medial knee OA were included in this study. Patients with symptomatic medial knee OA with articular cartilage lesions on the medial femoral condyle (MFC) or medial tibial plateau (MTP) were indicated to OWHTO, and with longitudinal or radial tears extended to peripheral lesions with mild meniscus degeneration without varus long‐leg alignment were included to medial meniscus repair, and with a flap tear and severe meniscus degeneration without varus long‐leg alignment were indicated to medial meniscus partial resection. The patients with an active knee infection, inflammatory diseases including rheumatoid arthritis, medial knee OA due to trauma complicated by ligamentous injuries such as anterior cruciate ligament injuries, and a history of previous surgeries were excluded. Among the 202 knees, 10 knees had previous anterior cruciate ligament reconstruction, 3 had previous medial meniscus repair and 11 had no preoperative long‐leg radiographs. Finally, 178 knees were included for statistical analysis in this study. The patient's age, sex and body mass index (BMI) were recorded (Table 1).

**Table 1:** Patient demographic data.

	Total	OWHTO	MM repair	MM partial resection
Number of cases	178	141	30	7
Age, years	57 [47–62]	58.0 [50.0–63.0]	46.5 [37.0–57.0]	44.0 [37.5–56.0]
Sex, male: female	76: 102	56: 85	16: 14	4: 3
Body mass index, kg/m^2^	25.5 [23.2–28.4]	25.5 [23.5–28.4]	25.5 [23.0–29.4]	24.0 [23.0–27.6]
Kellgren–Lawrence grade				
1	45	17	22	6
2	44	37	6	1
3	74	72	2	0
4	15	15	0	0
International Cartilage Repair Society grade on the medial femoral condyle				
0	16	0	13	3
1	12	5	7	0
2	27	18	6	3
3	48	44	4	0
4	75	74	0	1
International Cartilage Repair Society grade on the medial tibial plateau				
0	13	4	9	0
1	41	21	15	5
2	36	29	6	1
3	53	52	0	1
4	35	35	0	0

*Note*: Data are presented as medians [interquartile ranges].

Abbreviations: MM, medial meniscus; OWHTO, open wedge high tibial osteotomy.

### Radiographic evaluation

The preoperative anteroposterior view of the knee and long‐leg radiograph were obtained in the full weight‐bearing position. Kellgren–Lawrence grade was assessed by two orthopaedic surgeons (T.T. and E.S.) for interrater reliability and repeated at an interval of 1 month for intrarater reliability. With long‐leg radiographs, hip–knee–ankle angle (HKAA), mechanical lateral femoral angle (mLDFA), medial proximal tibial angle (MPTA) and joint line convergence angle (JLCA) were measured, with digital planning software (mediCAD; Hectec GmbH). The intrarater reliabilities [95% confidence interval, CI] of the Kellgren–Lawrence grade, HKAA, mLDFA, MPTA and JLCA were 0.912 [0.884–0.934], 0.939 [0.919–0.954], 0.925 [0.900–0.943], 0.877 [0.838–0.907] and 0.872 [0.832–0.903], respectively. Also, the interrater reliabilities of those parameters were 0.860 [0.813–0.896], 0.856 [0.809–0.962], 0.852 [0.807–0.932], 0.839 [0.782–0.881] and 0.820 [0.756–0.918], respectively.

### Magnetic resonance imaging evaluation

For evaluation of the condition of the medial meniscus, medial meniscus extrusion (MME) relative to the medial tibial margin was measured using preoperative magnetic resonance imaging in the previously reported method [7].

### Arthroscopic evaluation

The grade of cartilage degeneration at the MFC and the MTP was evaluated using the International Cartilage Research Society (ICRS) grading system [4] during surgery. The measurements were obtained by two orthopaedic surgeons (T.T. and E.S.) for interrater reliability and repeated at an interval of 1 month for intrarater reliability. The intrarater reliabilities [95% CI] of the ICRS grade on the MFC and the MTP were 0.905 [0.875–0.928] and 0.905 [0.875–0.928], respectively. Also, the interrater reliabilities of those parameters were 0.890 [0.855–0.917] and 0.912 [0.881–0.934], respectively.

### Statistical analysis

All statistical data were presented as medians [interquartile ranges]. The correlations between BMI, Kellgren–Lawrence grade, ICRS grade, and HKAA, mLDFA, MPTA and JLCA were evaluated using Spearman's correlation test respectively. Factors related to Kellgren–Lawrence grade were investigated using linear regression analysis, with Kellgren–Lawrence grade as the dependent variable, and age, sex, BMI, HKAA, mLDFA, MPTA and JLCA as independent variables. Factors related to ICRS grade were also assessed using linear regression analysis, with ICRS grade on the MFC and the MTP, respectively, as the dependent variable, and sex, BMI, HKAA, mLDFA, MPTA and JLCA as independent variables. Finally, receiver operating characteristic (ROC) curve analysis was performed to determine the optimal JLCA for each ICRS grade on the MFC and the MTP, respectively. All statistical analyses were performed using SPSS (version 29.0, SPSS, Inc.), and a *p* value ˂ 0.05 was considered statistically significant. An odds ratio with a 95% confidence interval that did not include 1 was considered statistically significant. A post hoc sample size analysis indicated that our series allowed the comparison of lower limb morphological parameters with a statistical power >80%.

## RESULTS

Seventy‐six males and 102 females were enroled in this study, and the median age was 57 [47–62] years. The median BMI was 25.5 kg/m^2^. The BMI was not correlated with HKAA, mLDFA, MPTA and JLCA by Spearman's correlation test.

### Relationship between joint line convergence angle and Kellgren–Lawrence grade

**Figure 1:**
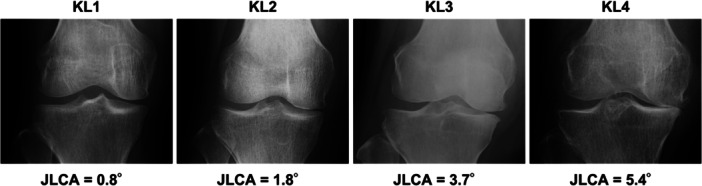
Representative radiographs of Kellgren–Lawrence grade and joint line convergence angle. JLCA, joint line convergence angle; KL, Kellgren–Lawrence grade.

**Figure 2:**
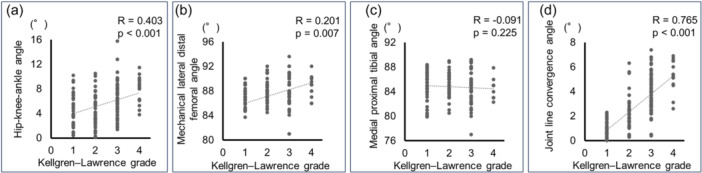
Relationship between Kellgren–Lawrence grade and hip‐knee‐ankle angle (a), mechanical lateral distal femoral angle (b), medial proximal tibial angle (c), and joint line convergence angle (d). The analysis was performed using Spearman's correlation test.

The JLCA values for each Kellgren–Lawrence grade from Grades 1 to 4 were 0.8° [0.5°–1.4°], 1.8° [1.5°–2.6°], 3.7° [2.8°–5.0°] and 5.4° [4.9°–6.5°], respectively (Table 2 and Figure 1). HKAA (*R* = 0.403, *p* < 0.001), mLDFA (*R* = 0.201, *p* = 0.007) and JLCA (*R* = 0.765, *p* < 0.001) were significantly correlated with Kellgren–Lawrence grade by Spearman's correlation test (Figure 2). Furthermore, age (*β* = 0.164, *p* = 0.002) and JLCA (*β* = 0.687, *p* < 0.001) were significantly associated with Kellgren–Lawrence grade by linear regression analysis, but not HKAA, mLDFA or MPTA (Table 3).

**Table 2:** The median value of each parameter by Kellgren–Lawrence grade.

Kellgren–Lawrence grade	1	2	3	4
Hip–knee–ankle angle	3.5 [2.1–6.3]	4.1 [2.6–6.6]	6.1 [4.6–7.9]	8.2 [6.1–9.3]
Mechanical lateral distal femoral angle	87.5 [86.0–88.4]	88.0 [87.0–89.0]	88.0 [87.0–89.3]	89.0 [87.5–89.8]
Medial proximal tibial angle	85.4 [83.5–87.0]	85.3 [84.0–86.5]	85.0 [83.0–86.0]	85.0 [83.5–85.0]
Joint line convergence angle	0.8 [0.5–1.4]	1.8 [1.5–2.6]	3.7 [2.8–5.0]	5.4 [4.9–6.5]

*Note*: Data are presented as medians [interquartile ranges].

**Table 3:** Related factors for Kellgren–Lawrence grade.

	*β*	*p* Value	95% CI
Age	0.164	0.002	0.01, 0.02
Sex	0.016	0.760	−0.16, 0.22
Body mass index	0.067	0.183	−0.01, 0.04
Hip–knee–ankle angle	−0.029	0.716	−0.06, 0.04
Mechanical lateral distal femoral angle	0.018	0.773	−0.05, 0.07
Medial proximal tibial angle	−0.093	0.185	−0.09, 0.02
Joint line convergence angle	0.687	<0.001	0.28, 0.40

*Note*: The analysis was performed by linear regression analysis. The dependent variable was the Kellgren–Lawrence grade.

Abbreviation: CI, confidence interval.

## RELATIONSHIP BETWEEN JOINT LINE CONVERGENCE ANGLE AND MEDIAL MENISCUS EXTRUSION

**Figure 3:**
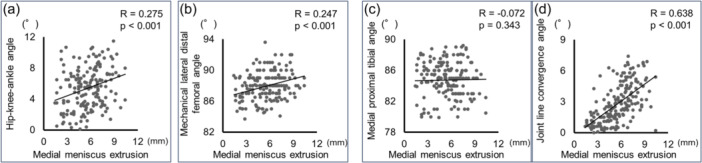
Relationship between medial meniscus extrusion and hip‐knee‐ankle angle (a), mechanical lateral distal femoral angle (b), medial proximal tibial angle (c), and joint line convergence angle (d). The analysis was performed using Spearman's correlation test.

The MME was significantly correlated with HKAA (*R* = 0.275, *p* < 0.001), mLDFA (*R* = 0.247, *p* < 0.001) and JLCA (*R* = 0.638, *p* < 0.001) by Spearman's correlation test (Figure 3).

### Relationship between joint line convergence angle and cartilage degeneration

**Figure 4:**
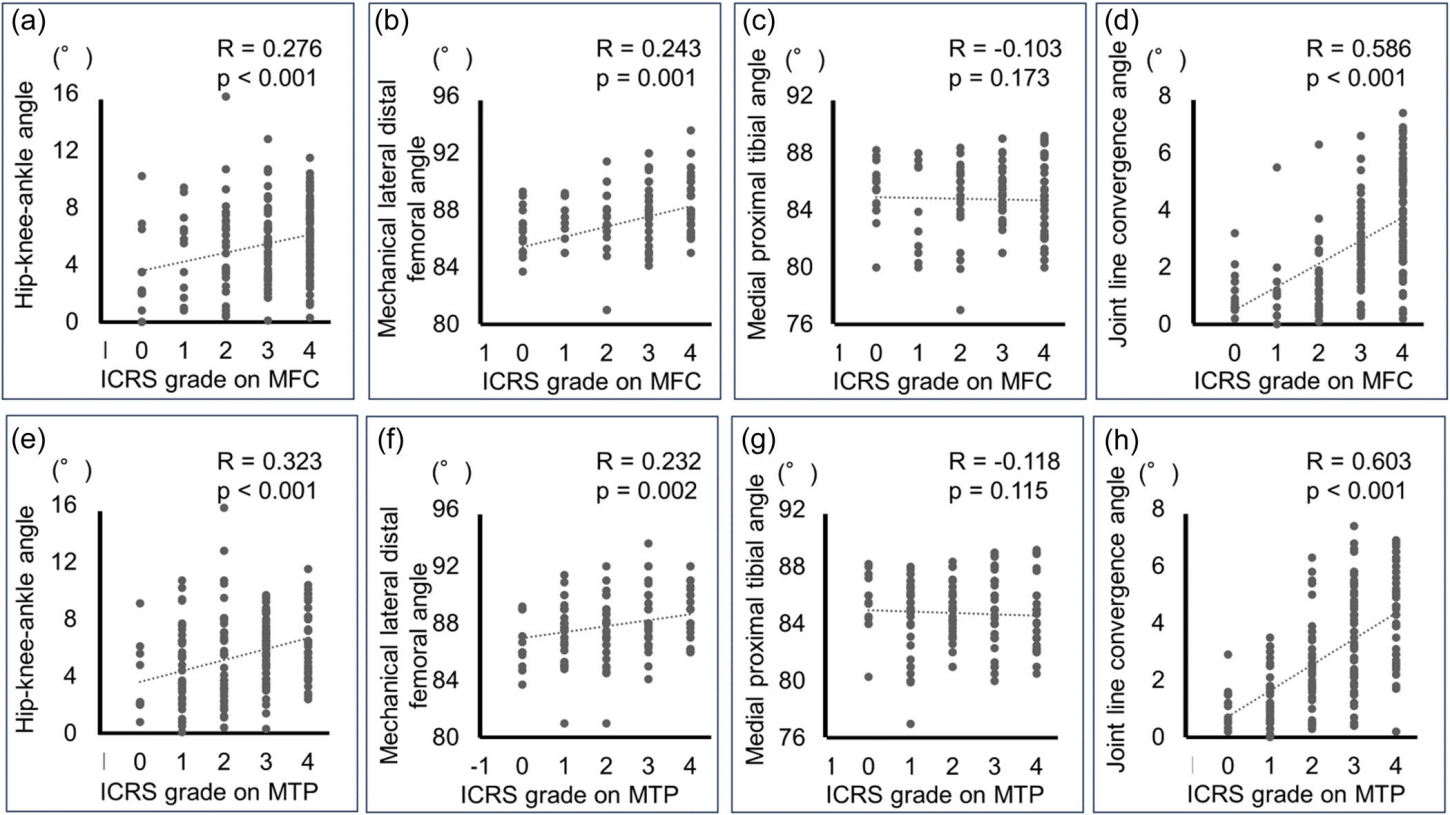
Relationship between International Cartilage Research Society grade and hip‐knee‐ankle angle (a, e), mechanical lateral distal femoral angle (b, f), medial proximal tibial angle (c, g), and joint line convergence angle (d, h). The analysis was performed using Spearman's correlation test. ICRS, International Cartilage Repair Society grade; MFC, medial femoral condyle; MTP, medial tibial plateau.

**Figure 5:**
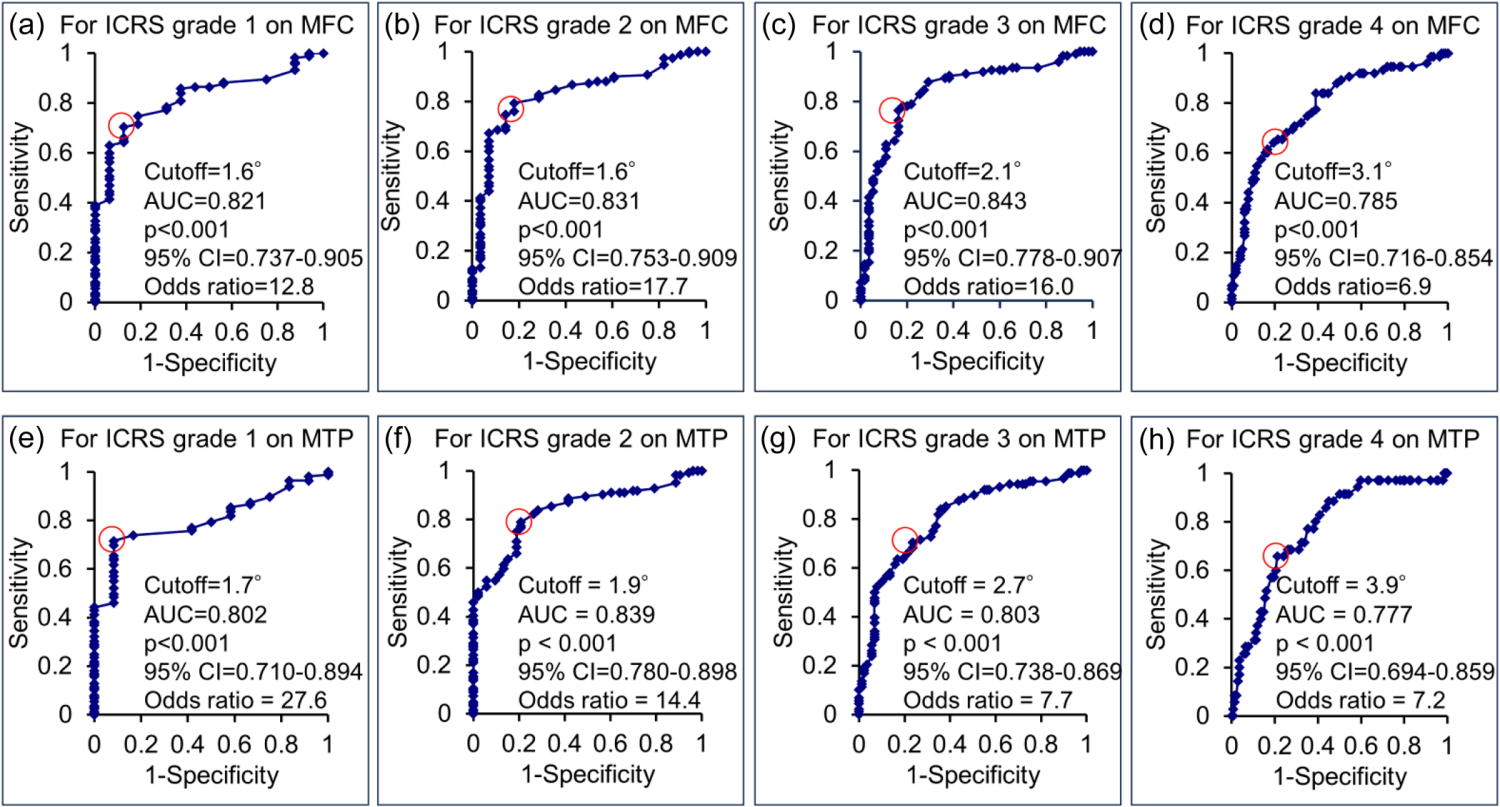
The cut‐off value of the joint line convergence angle for ICRS grade 1 (a, e), grade 2 (b, f), grade 3 (c, g), and grade 4 (d, h). The analysis was performed using receiver operating characteristic analysis. AUC, area under the curve; CI, confidence interval; ICRS, International Cartilage Repair Society grade; JLCA, joint line convergence angle; MFC, medial femoral condyle; MTP, medial tibial plateau.

The JLCA values for each ICRS grade from Grades 0 to 4 on the MFC were 0.8° [0.6°–1.5°], 1.1° [0.5°–1.5°], 1.2° [0.6°–2.2°], 2.7° [1.8°–3.5°] and 4.0° [2.5°–5.4°], respectively (Table 4). The HKAA (*R* = 0.276, *p* < 0.001), mLDFA (*R* = 0.243, *p* = 0.001) and JLCA (*R* = 0.586, *p* < 0.001) were significantly correlated with ICRS grade on the MFC by Spearman's correlation test (Figure 4a–d). Furthermore, linear regression analysis revealed that age (*β* = 0.310, *p* < 0.001), mLDFA (*β* = 0.137, *p* = 0.026) and JLCA (*β* = 0.477, *p* < 0.001) were significantly associated with ICRS grade on the MFC (Table 5). The ROC analysis determined cut‐off values for JLCA at 1.6° for ICRS Grade ˃1 (*p* < 0.001; Figure 5a), 1.6° for Grade ˃2 (*p* < 0.001; Figure 5b), 2.1° for Grade ˃3 (*p* < 0.001; Figure 5c) and 3.1° for Grade 4 (*p* < 0.001; Figure 5d).

**Table 4:** The median value of each parameter by ICRS grade.

ICRS grade on the MFC	0	1	2	3	4
Hip–knee–ankle angle	2.1 [1.7–3.5]	5.6 [2.2–6.7]	5.3 [3.2–7.0]	5.4 [3.2–6.5]	6.1 [4.2–8.1]
Mechanical lateral distal femoral angle	86.3 [85.1–87.3]	87.5 [86.5–89.0]	87.6 [86.7–89.0]	88.0 [87.6–89.6]	88.0 [87.0–89.2]
Medial proximal tibial angle	85.5 [84.3–86.3]	85.5 [84.3–86.3]	85.5 [83.9–86.5]	85.0 [84.0–86.0]	85.0 [83.0–86.0]
Joint line convergence angle	0.8 [0.6–1.5]	1.1 [0.5–1.3]	1.2 [0.6–2.2]	2.7 [1.8–3.5]	4.0 [2.5–5.4]

ICRS grade on the MTP	0	1	2	3	4
Hip–knee–ankle angle	2.1 [0.8–4.8]	4.4 [2.9–6.5]	5.0 [2.8–7.2]	6.1 [3.1–7.4]	6.3 [4.4–8.2]
Mechanical lateral distal femoral angle	86.0 [85.8–89.0]	88.0 [86.5–89.0]	88.0 [86.8–89.0]	88.0 [87.0–89.0]	88.0 [87.9–90.0]
Medial proximal tibial angle	85.5 [84.4–87.2]	85.5 [83.9–86.5]	85.0 [83.4–86.1]	85.0 [84.0–86.0]	84.7 [82.8–85.2]
Joint line convergence angle	1.1 [0.5–1.5]	1.1 [0.6–1.8]	2.5 [1.6–3.6]	3.3 [2.2–3.7]	4.5 [2.9–5.7]

*Note*: Data are presented as medians [interquartile ranges].

Abbreviations: ICRS, International Cartilage Repair Society; MFC, medial femoral condyle; MTP, medial tibial plateau.

**Table 5:** Related factors for the International Cartilage Repair Society grade on the medial femoral condyle.

	The analysis focusing on the MFC			The analysis focusing on the MTP		
	*β*	*p* Value	95% CI	*β*	*p* Value	95% CI
Age	0.310	<0.001	0.02, 0.05	0.121	0.052	0.00, 0.03
Sex	0.001	0.993	−0.30, 0.30	−0.031	0.606	−0.37, 0.22
Body mass index	0.050	0.387	−0.02, 0.06	−0.018	0.756	−0.05, 0.03
Hip–knee–ankle angle	0.106	0.064	0.00, 0.10	0.152	0.010	0.02, 0.12
Mechanical lateral distal femoral angle	0.137	0.026	0.01, 0.17	0.125	0.048	0.00, 0.16
Medial proximal tibial angle	−0.107	0.070	−0.11, 0.00	−0.080	0.186	−0.10, 0.02
Joint line convergence angle	0.477	<0.001	0.24, 0.40	0.561	<0.001	0.28, 0.44

*Note*: The analysis was performed by linear regression analysis. The dependent variable was the International Cartilage Repair Society grade.

Abbreviations: CI, confidence interval; MFC, medial femoral condyle; MTP, medial tibial plateau.

The JLCA values for each ICRS grade from Grades 0 to 4 on the MTP were 1.1° [0.5°–1.5°], 1.1° [0.6°–1.8]°, 2.5° [1.6°–3.6°], 3.3° [2.2°–3.7°] and 4.5° [2.9°–5.7°], respectively (Table 4). The HKAA (*R* = 0.323, *p* < 0.001), mLDFA (*R* = 0.232, *p* = 0.002) and JLCA (*R* = 0.603, *p* < 0.001) were significantly correlated with ICRS grade on the MTP by Spearman's correlation test (Figure 4e–h). Furthermore, linear regression analysis revealed that HKAA (*β* = 0.152, *p* = 0.010), mLDFA (*β* = 0.125, *p* = 0.048) and JLCA (*β* = 0.561, *p* < 0.001) were significantly associated with ICRS grade on the MTP (Table 5). Furthermore, ROC analysis determined cut‐off values for JLCA at 1.7° for ICRS Grade ˃1 (*p* < 0.001; Figure 5e), 1.9° for Grade ˃2 (*p* < 0.001; Figure 5f), 2.7° for Grade ˃3 (*p* < 0.001; Figure 5g) and 3.9° for Grade 4 (*p* < 0.001; Figure 5h).

## DISCUSSION

The most important finding of this study was that the JLCA was related to Kellgren–Lawrence grade, MME and cartilage degeneration in patients with medial knee OA. Additionally, the JLCA could predict the degree of cartilage degeneration.

Its diagnosis is generally confirmed through physical examinations and radiographs, with the Kellgren–Lawrence grading system that is well established for assessing knee OA progression [13, 28, 30]. The Kellgren–Lawrence grading is based on the number of osteophytes and the joint space narrowing [8, 13, 30]. However, this assessment is not quantitative and does not consider long‐leg alignment, as it relies on an anteroposterior view of the knee.

The JLCA is measured as the angle between the tangent line at the most distal ends of both femoral condyles and the subchondral plateau of the tibial plateau in the anteroposterior view of a full‐weight bearing long‐leg radiograph [15, 19, 20, 26, 29]. It also reflects soft tissue contracture or laxity around the knee [15, 24, 29]. Soft tissue laxity affects intraarticular pressure [1, 9] and exhibits variations between sexes [18]. Moser et al. [23] assessed the coronal alignment of the non‐OA knees, reporting a mean JLCA of 0.47 ± 0.98° in male and 1.9 ± 1.4° in female patients.

Regarding JLCA with knee OA, Stotter et al. [32] evaluated varus alignment in patients who underwent medial OWHTO, revealing a mean preoperative JLCA of 2.2 ± 2.0° in artificial intelligence‐based analyses and 2.0 ± 1.8° in manual measurements. Behrendt et al. [3] reported preoperative JLCA correction in varus knee correction osteotomy, reporting a mean preoperative JLCA of 2.5° ± 1.8°. Glowalla et al. [6] reported a preoperative JLCA of 3.7° in the full weight‐bearing position and 3.0° in the nonweight‐bearing position in patients with advanced knee OA treated with TKA. Hence, JLCA was considered to increase with the severity of knee OA. Recently, Mabrouk et al. [19] reported a correlation between JLCA and Kellgren–Lawrence grade.

Regarding the relationship between JLCA and meniscus status, Kozaki et al. [14] using finite element analysis reported that medial MME is associated with increased stress loading on the medial knee compartment, exacerbated by a larger JLCA. Other studies have focused on the effect of the JLCA on correction after medial OWHTO. In addition, Goto et al. [7] reported that HKAA, MPTA and JLCA were significant independent factors of MME distance. Similarly in this study, the MME distance was significantly correlated with JLCA. Moreover, Lee et al. [16] reported that preoperative and postoperative JLCA are associated with postoperative medial joint space widening. Decompression of the medial knee compartment after medial OWHTO can improve the flow of synovial fluid feeding the articular cartilage and benefit cartilage metabolism [22]. These reports also suggest that JLCA may increase with meniscus dysfunction associated with OA severity, followed by loading of the articular cartilage of the medial femorotibial joint and progression of cartilage degeneration.

In this study, preoperative JLCA was associated with OA severity and cartilage degenerations in the medial knee compartment. JLCA emerged as a potential predictor of the degree of cartilage degeneration, thus providing a quantitative assessment of the OA severity in the medial knee compartment. This information may facilitate orthopaedic surgeons in making decisions regarding indications for surgery.

This study had certain limitations. First, this was a retrospective, nonrandomised, cross‐sectional study. A long‐term longitudinal evaluation may provide more evident results on JLCA and severity of knee OA. Second, this study included patients with medial knee OA who had undergone arthroscopic surgery. Therefore, end‐stage OA requiring TKA was not included. The patients with Kellgren–Lawrence Grade 4 in this study were not included in cases with subchondral bone wear in the MTP, which may have resulted in no association between MPTA and Kellgren–Lawrence grade or ICRS grade. Finally, the present study showed that JLCA is associated with the severity of medial knee OA, so it is predicted that JLCA is associated with patient‐reported outcomes. This should also be investigated in further study.

## CONCLUSIONS

The JLCA, reflecting radiological severity, meniscus status and cartilage lesion, was the most associated alignment parameter in the severity of medial knee OA. The JLCA may be beneficial for quantitative assessment of medial knee OA.

## AUTHOR CONTRIBUTIONS

Takahiro Tsushima was responsible for the organisation and coordination of this study. All authors contributed to the management of this study as well as the acquisition, analysis and interpretation of the data. All the authors have approved the manuscript for publication.

## CONFLICT OF INTEREST STATEMENT

The authors declare no conflict of interest.

## ETHICS STATEMENT

This study was approved by the ethics committee of our institution (No. 2019‐596‐1). Informed consent was obtained from all study participants.

## Data Availability

The data that support the findings of this study are openly available.
